# Indiscriminate Males: Mating Behaviour of a Marine Snail Compromised by a Sexual Conflict?

**DOI:** 10.1371/journal.pone.0012005

**Published:** 2010-08-09

**Authors:** Kerstin Johannesson, Sara H. Saltin, Iris Duranovic, Jon N. Havenhand, Per R. Jonsson

**Affiliations:** Department of Marine Ecology - Tjärnö, University of Gothenburg, Strömstad, Sweden; University of Otago, New Zealand

## Abstract

**Background:**

In promiscuous species, male fitness is expected to increase with repeated matings in an open-ended fashion (thereby increasing number of partners or probability of paternity) whereas female fitness should level out at some optimal number of copulations when direct and indirect benefits still outweigh the costs of courtship and copulation. After this fitness peak, additional copulations would incur female fitness costs and be under opposing selection. Hence, a sexual conflict over mating frequency may evolve in species where females are forced to engage in costly matings. Under such circumstance, if females could avoid male detection, significant fitness benefits from such avoidance strategies would be predicted.

**Methodology/Principal Findings:**

Among four *Littorina* species, one lives at very much higher densities and has a longer mating season than the other three species. Using video records of snail behaviour in a laboratory arena we show that males of the low-density species discriminate among male and female mucous trails, trailing females for copulations. In the high-density species, however, males fail to discriminate between male and female trails, not because males are unable to identify female trails (which we show using heterospecific females), but because females do not, as the other species, add a gender-specific cue to their trail.

**Conclusions/Significance:**

We conclude that there is likely a sexual conflict over mating frequency in the high-density species (*L. saxatilis*) owing to females most likely being less sperm-limited in this species. This has favoured the evolution of females that permanently or optionally do not release a cue in the mucus to decrease excessive and costly matings resulting in unusually high frequencies of male-male copulating attempts in the wild. This is one of few examples of masking gender identity to obtain fewer matings.

## Introduction

For many species the number of matings that maximizes fitness differs between the sexes. Male fitness is generally believed to increase with the number of matings, whereas polyandry is favoured by selection under conditions such as depletion of sperm in a female's reproductive tract or indirect genetic benefits via sperm competition or cryptic female choice [1], [2]. After an initial increase in fitness with number of copulations, female fitness starts to decline when costs of mating exceed gains from receiving additional sperm [3]–[6]. Thus as a consequence of this sexual conflict females may evolve measures to avoid the costs of excessive matings while males remain selected for maximizing paternity through repeated mating [7]–[9]. This situation may arise, for example, in populations of promiscuous species that live at high densities, store sperm over several ovarian cycles, have long mating seasons, but modest egg production compared to other species. This gives males many mating opportunities, while females risk decreased fitness from superfluous costly matings. In this study we test this prediction using marine intertidal periwinkle species of the genus *Littorina* which have separate sexes and are typically highly promiscuous [10]–[12]. However, the opportunity for males to transfer sperm (mate), and female requirements for sperm, vary among the four species chosen for this study, owing to differences in length of mating season, densities of snails and overall egg production ([13] and data presented herein).

**Figure 1:**
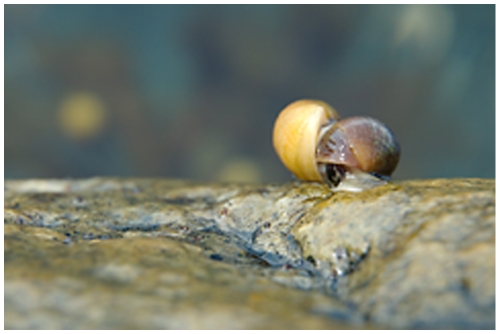
A copulating pair of *Littorina* (*Littorina saxatilis* of Swedish S ecotype). The female is attached to the substratum while the male is positioned on the right-hand side of her shell, inserting his penis under her shell. Photo: Patrik Larsson.

Male-female pairing in snails is preceded by mucus-trail tracking, predominantly by males [12]. The accuracy of this behaviour in terms of conspecific, heterosexual trailing is likely to be under selection, since crawling in snails requires production of substantial amounts of energetically-expensive pedal mucus [14], [15]. Once a partner is located, a male littorinid snail mounts the female and after a counter-clockwise movement on the shell, he stops at the right-hand side of the shell and inserts the penis under the shell of the partner (Fig. 1). Only rarely have females been observed to actively reject a mounting male [11]. Sperm is transferred through ciliary actions in a groove along the penis (Buckland-Nicks pers. commun.), and copulations typically last for 10–30 min [11], [16]. During the copulation the female effectively “carries” the male, incurring a cost to the mating pair as risk of predation increases for mating pairs compared to single individuals [17]. Dislodgement by waves is an additional selective pressure for intertidal snails, as being removed from the intertidal increases risk of predation by crabs and fishes [18], [19]. Risk of dislodgement is determined by the hydrodynamic drag (proportional to the cross-sectional area of the snail) and attachment strength (proportional to the foot area), and we therefore hypothesis that a copulating pair should have considerably greater risk of dislodgement because cross-sectional area is doubled with no change in attachment strength.

It has been suggested that among gastropods in general, males identify (and are attracted to) partners prior to copulation through specific chemical cues added to the trail mucus by the female [20]–[22]. Earlier observations suggest that female trailing by males occurs in the periwinkle species *Littorina littorea* [21], but appears to be remarkably random with respect to sex in male *L. saxatilis* [12]. This may be due to (*i*) male lacking the capacity to differentiate between males and females, and/or (*ii*) the evolution of sexual mimicry in females, avoiding the costs of superfluous copulations. Here, we experimentally test these two hypotheses. Furthermore, we hypothesize that the difference in male behaviour between *L. littorea* and *L. saxatilis* is a consequence of a potentially more pronounced sexual conflict over mating frequency in the latter species, owing to its much higher density and prolonged mating-season. To test this hypothesis we make the additional prediction that gender-specific trail following would be in the norm in two other littorinid species, *L. obtusata* and *L. fabalis*, that share the restricted mating season and low density with *L. littorea*, although their yearly egg production is similar to *L. saxatilis* [13].

## Materials and Methods

### Estimates of population densities

Population density of each of the four species was estimated by sampling 6–10 typical habitats of each species in the area of investigation (the archipelago around the island Tjärnö on the Swedish west coast, 58°53′N, 11°8′E). In each site, 1 m^2^ large areas were investigated. First we harvested all macroalgae and these were brought back to the laboratory, and secondly we carefully searched the rock surface and picked all visible snails (>1–2 mm). In the laboratory, macroalgae were put in tanks with freshwater causing snails in the algal canopy to drop to the bottom of the tank facilitating collection. Snails that inhabit the macroalgal canopy (in this study *L. fabalis* and *L. obtusata*) experience the area of the algal thalli as their total available surface. Hence for these two species we estimated population densities in relation to total macroalgal surface area (calculated from the surface area and wet-weight of a subsample of algae, and the total weight of the sample). In contrast, for *L. saxatilis* and *L. littorea* we estimated 3-dimensional surface area from the area of the boulders or rocky substrates (including crevices etc.). Total thallus area of 1 m^2^ 100% cover of *Ascophyllum nodosum* was estimated to 24 m^2^, and the corresponding area for *Fucus vesiculosus* was 33 m^2^.

### Experimental tests of female costs of mating

In addition to increased predation risk [17] a main cost of mating is likely to be the increased risk of dislodgement of a copulating pair compared to the risk of a single snail, as hydrodynamic drag will be roughly doubled for a copulating pair while attachment strength remains that of a single snail. In a laboratory experiment we glued male *L. saxatilis* shells in copulating positions onto the females of the same species (to simulate mounting males) and compared the risk of dislodgement with that of single females. In each run we allowed five females with and five females without glued male shells attach themselves to a PVC platform (40×30 cm). The platform was then towed at constant speed along the bottom of a 6 m tank. For each tow the number of detached females of each type was recorded. The platform was towed repeatedly at increasing speeds corresponding to the range found in breaking waves with wave heights less than 1 m [23]. Using results from 10 replicate tows (new snails in each), the difference in flow speed needed to remove half the number of snails was compared between females with and without a glued male shell. This experiment was complemented by a release-recapture of artificially ‘paired’ versus single females in the wild; 29 females with and 28 without glued male shells were marked with paint and released on a natural rocky shore. Recapture took place a week later.

### Trail-following experiments

Snails of *Littorina littorea*, *L. fabalis*, *L. obtusata* and *L. saxatilis* (Swedish cliff ecotype, E morph, [24]) were sampled at two sites each along the shores of three small islands (Saltö, Långholmen and Lökholmen; in the vicinity of Tjärnö in Sweden). *Littorina saxatilis* is an exceptionally polymorphic species [13] and in addition to using the Swedish E ecotype we compared our results for this ecotype with the behaviour of the Swedish S ecotype (from two localities on Saltö) that are morphologically and ecologically very different and live in different microhabitats but at similarly high densities [24]. Finally, we analysed male behaviour also with *L. saxatilis* from Spain sampled in exposed low-shore habitats in Galicia, (42°6′N, 8°53′W). Although considered the same species, Spanish and Swedish *L. saxatilis* are very distantly related and have evolved separately for more than 100,000 years (Panova et al. subm.), but again densities, reproductive season and reproductive modes are very similar.

In a first series of tests we studied male behaviour in each of the four species and the three different *L. saxatilis* populations separately. In one run, five males and five females were allowed to move freely in an arena (<0.25 m^2^) and their movements were recorded with digital video over a period of 15 minutes. For each group at least 14 replicate runs (new snails for each run) testing trail-following behaviour were performed. The surface of the arena was wetted with seawater and rinsed carefully between each run. Spatial excursions and extent of trail-following by male conspecifics were measured from the video using computerised motion analysis (CellTrak for Windows, Motion Analysis Corp.). Male movements were defined as trail-following when following occurred for more than 3 snail diameters. All other paths and paths along the edges of the arena were excluded from our analyses. Hence from each run we received the total distance a male tracker followed male trails and female trails, respectively. As the level of replication in our experiments was a run, we pooled the results from all 5 males from each run by using the total tracking distances of all 5 males. As we did not include data for males tracking their own trails, we randomly removed the data from one female in each run (avoiding bias but maintaining gender balance during the experiment).

During the 15 minutes experiments, several males did not encounter any trail to follow, and in many cases we only recorded one trail-following event per run. Our main experiment was therefore complemented by a study in which we tested male tracking behaviour in one of the groups (E ecotype *L. saxatilis*) over a longer period of time (60 instead of 15 minutes) in order to remove the possibility that trial duration constrained our probability of detecting sex-biased male trailing. This study was performed in the same way as the experiments described above, with the exception that we used a larger arena (to delay snails reaching the edge as much as possible).

To specifically test the hypothesis that male *L. saxatilis* had lost their capacity to identify female trails, we did a separate series of experiments in which we used male *L. saxatilis* as trackers and males and females of either *L. saxatilis* or *L. fabalis* as markers. In random order we performed 20 replicate runs in total for the combination with male and female *L. saxatilis* as markers, and 20 with male and female *L. fabalis* as markers (in both using male *L. saxatilis* as tracker). The trail-following of the trackers were analysed in the same way as described above. Also in this experiments, snails were from different localities at the island of Saltö.

In all the trail-following experiments we used a binomial test to discriminate between two possible outcomes of the experiments; *H_0_* – that males followed male and female trails for equal distances (or followed male distances longer than female distances); *H_1_* – that males followed female mucous trails for longer distances than they followed other male trails. Thus, we test the directional prediction that males followed females for longer and therefore apply a one-tailed test.

## Results

### Snail densities

Combining new data for snail densities in their natural habitat with literature data showed that densities of *L. saxatilis* were >100× higher than for the other species (Table 1), and therefore expected male-female encounter rates would be two orders of magnitude greater in this species. In addition, a longer mating season further increases the total number of matings per reproductive season in female *L. saxatilis* in comparison to the other species (Table 1). Hence, female *L. saxatilis* are far less likely to be sperm-limited than females of any of the other species.

**Table 1:** Life-history traits in four species of *Littorina*.

Species	Reproductive season	Number of eggs per year	Snail density^1^
*L. saxatilis^2^*	Year round^3^	200^4^	280
*L. littorea*	February–June^3^	110,000^5^	2.3
*L. fabalis*	Spring-Autumn^3^	>600^4^	1.4
*L. obtusata*	March–September^3^	>500^4^	1.1

Densities for *L. saxatilis* are indicated for the Swedish cliff ecotype (E). Data on reproductive seasons are from [13], and numbers of eggs per year are from unpublished studies by KJ except for *L. littorea* where data are from Buschbaum & Reise [37]. Snail density estimates are from typical habitats of each species taken into account the surface area of the substratum, see text for details.

### Cost of mating

Our laboratory trials showed that females with a ‘pairing male’ required significantly lower water speed to dislodge the pair as opposed to single females. The average water speed required to detach 50% of the females with an attached male was significantly lower than that for females without an attached male (0.6 m s^−1^ *vs* 1.2 m s^−1^; 1-factor ANOVA, two-tailed, *F*_1,9_ = 5.77, *P* = 0.00027).

In the accompanying field experiment, we recovered significantly fewer females with attached male shells (3 out of 29) than females without male shells (12 out of 28) (χ^2^ = 4.6, *df* = 1, *P*<0.05), indicating a survival difference between the two experimental groups of females.

### Trail following

**Figure 2:**
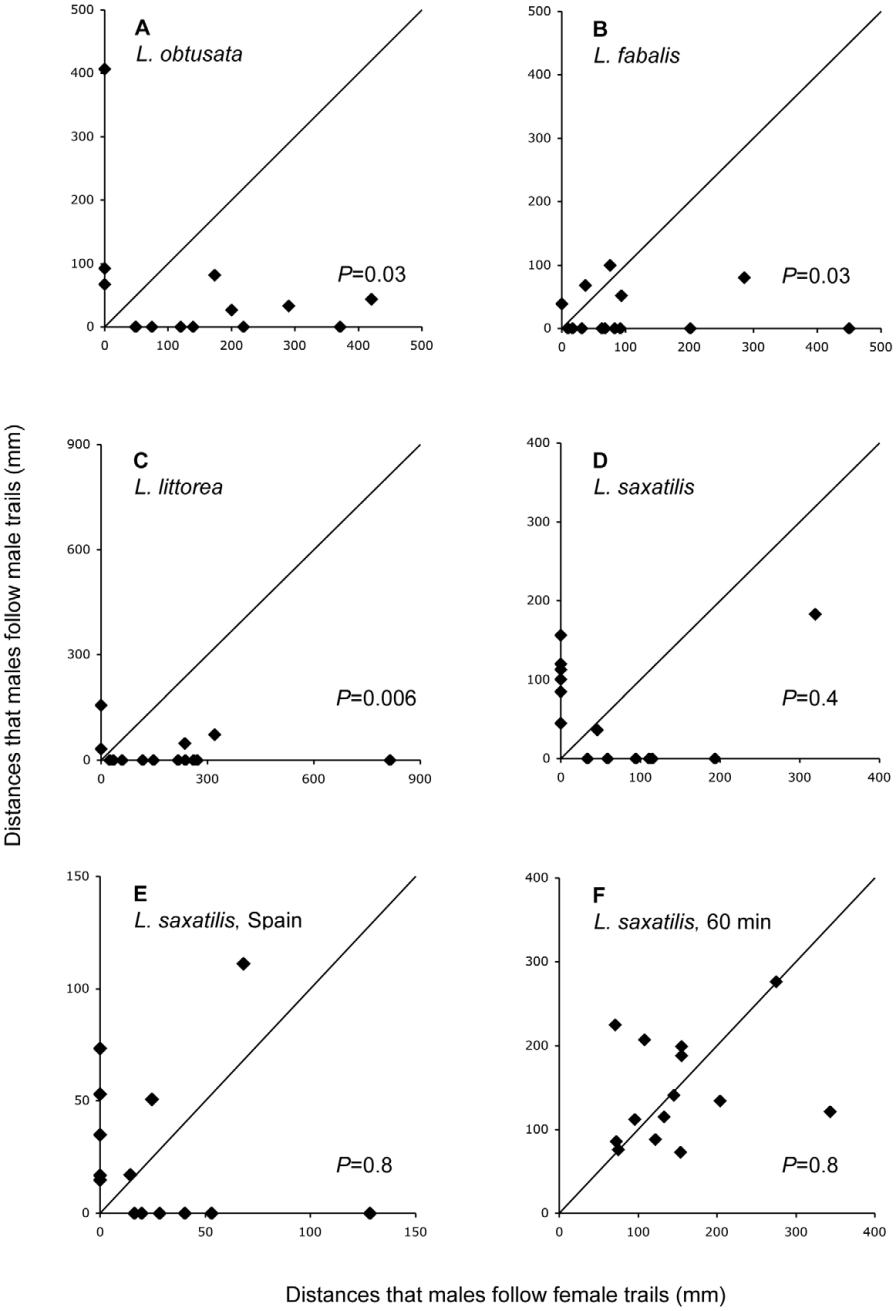
Male tracking females and other males in four species of *Littorina*. Distances that male trackers followed males (y-axis) and females (x-axis) in four different species of *Littorina*. In *L. saxatilis* two geographically separated populations (Sweden E-ecotype, and Spain SU-ecotype) are analysed. Each point represents the total distance of trail-following of 5 males of one replicate run (15 min, or 60 min in F). The diagonal indicate the expectation of equal tracking distances of female and male markers. One-tailed *P*-values of binomial tests are indicated.

Males of all species identified the polarity of the trail and followed the trail in the direction of the marker snail in about 90% of the cases (Table 2). In the low-density species *L. littorea*, *L. obtusata* and *L. fabalis* males were also significantly more likely to follow female mucous trails than male mucous trails (binomial test, *P* = 0.006–0.029; Fig. 2A–C). Although a few males of these three species were observed to follow trails of other males (points along the y-axis in Fig. 2), the great majority of males exclusively followed female trails, or followed female trails for much longer distances than male trails (points along or close to the x-axis in Fig. 2). In contrast, we found no evidence that male *L. saxatilis* could discriminate between the mucous trail of females and males, and these results were consistent for males from both the Swedish and the Spanish populations of this species (binomial test, *P* = 0.4 and *P* = 0.8; Fig. 2D–E) and for Swedish S ecotypes (*P* = 0.6; results not shown). In the longer-duration (60 min) runs, there was still no deviation from random trailing with respect to sex (Fig. 2F).

**Table 2:** Polarity of trail-following in male *Littorina*.

		Frequency of tracks (%)	
Species	N	Positive	Negative
*L. obtusata*	36	89	11
*L. fabalis*	56	89	11
*L. littorea*	50	92	8
*L. saxatilis*	24	100	0

**Figure 3:**
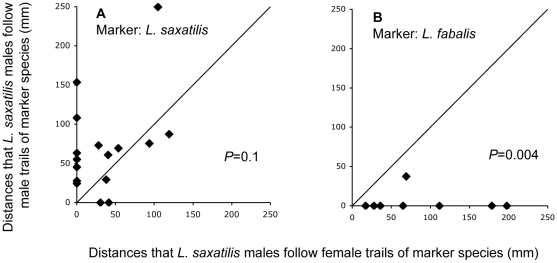
*Littorina saxatilis* males tracking males and females of their own or an other species (*L. fabalis*). (A) Distances that males of *L. saxatilis* followed males and females of their own species. (B) Distances that males of *L. saxatilis* followed males and females of *L. fabalis*. Experimental conditions and statistical tests as in Figure 1.

When we used *L. fabalis* as marker females the overall extent of tracking by *L. saxatilis* males was slightly less pronounced; tracking in 8 of 20 runs compared to 16 of 20 for the conspecific runs, which may be expected for a heterospecific comparison. Remarkably, however, in all runs where *L. fabalis* trails were followed, male *L. saxatilis* followed female trails for longer distances than male trails (binomial test, *P* = 0.004) while in the same experiment, *L. saxatilis* males, as before, did not discriminate between male and female trails of their own species (*P* = 0.1; Fig. 3). This shows that male *L. saxatilis* have the capacity to detect, and show a preference for, a female-specific mucous cue, and strongly suggests that this mucous cue is absent in female *L. saxatilis*.

## Discussion

Our results show that under similar experimental densities male trail-following behaviour of the high-density *L. saxatilis* is different from that of the related but naturally low-density periwinkle species, *L. littorea*, *L. fabalis* and *L. obtusata*. Indeed, results obtained in five independent experiments including geographically separated populations and different ecotypes, show that males of *L. saxatilis* can not discriminate between female and male mucous trails when tracking snails of their own species, whereas males of the other three species are able to identify female trails. However, male *L. saxatilis* can distinguish between male and female trails of another species, *L. fabalis*, and have thus not lost their ability to track females using a gender-specific mucous cue. This suggests that essentially the same female identification cue is shared among species and that female *L. saxatilis* have either lost this cue completely, or can optionally remove the cue when densities of mates are high. Although we have not identified the specific cue, a chemical cue that females add to their mucus and that attracts males seems quite likely, and such a cue is shown to be present in a freshwater snail [25]. Notably, in an earlier study we observed that male *L. saxatilis* were indeed able to discriminate between female *L. saxatilis* of different ecotypes, resulting in assortative mating [26]. Also in this study males did not discriminate between male and female trails, but data were only evaluated for male-female trackings.

The consequence of the different trail-following behaviours are readily observed in the field: in general, numbers of males mounting other males and juveniles are low (0–7%) in species of littorinids including tropical species distantly related to *L. saxatilis* [11], [27], [28], but *L. saxatilis* is an obvious exception with much higher rates of male-male or male-juvenile mating attempts (30–40%) [10]–[12]. Collectively, these data strongly suggest that both a female-specific cue added to the female mucous trail, and the capacity of the males to identify this cue, are ancestral traits among littorinid snails but that the gender cue is permanently lost or optionally removed at high densities in female *L. saxatilis*. As *L. saxatilis* is the most derived species in a phylogeny including the four littorinids of this study [29], there are 16 possible combinations with these four species each having or not having this particular character state. Therefore, there is only one chance in 16 that loss or optional regulation of this cue in female *L. saxatilis* is a result of pure chance, while it being a novel state (autapomorphy) seems much more likely.

Females that do not release a cue decrease the number of matings by producing a gender-neutral trail, and such a strategy would likely be favoured by selection if females are not sperm-limited and if costs of mating are substantial. Sperm-limitation seems most unlikely in *L. saxatilis*; much higher densities than in the other three littorinid species and longer, almost year-round, mating season, give excessive opportunities to mate. Recent data show that each female simultaneously carry offspring sired by 10–20 males [30], [31] supporting the prediction that female *L. saxatilis* are not sperm-limited. Indeed, such a high level of polyandry is hard to explain from the perspective of increased female fitness and we have earlier suggested this to be a consequence of convenience polyandry [31]. At the same time cost of mating is likely to be substantial as predation risk increases during mating, both directly through increased susceptibility to predators [17], and indirectly through increased risk of snails being dislodged to the more predator-rich subtidal [this study and 18], [19]. Hence mating costs would select for mechanisms in the female to avoid both male harassment and excessive matings, as a complement to convenience mating.

Costly matings may potentially be a problem also for male *L. saxatilis* and select for males that restrict the number of matings. As males of littorinids, however, provide no parental care, adding to the number of life-time matings is their only way to increase life-time fitness. Hence males would be prepared to take larger risks compared to females and strive to mate as frequently as possible despite these costs.

Is it necessary for males to track females in high-density populations such as *L. saxatilis*, when a male is likely to encounter a high number of females anyhow? As a male that practice mucus-tracking uses both time and energy during tracking and during mounting the partner (male-male copulation attempts are disrupted when the mating male tries to insert the penis under the shell of the other male [11]), a male that can avoid tracking other males, or juveniles, will be at an advantage over other males. Indeed our results showed that male *L. saxatilis* practice female tracking if encountering trails of males and females of another species (*L. fabalis*), and this indicates that male *L. saxatilis* still retain the capacity to discriminate between male and female trails if the female cue is present.

Why do not females produce more eggs and/or shorten the mating period to either match the high availability of sperm, or avoid male harassment by not being year-round receptive? In *L. saxatilis* egg production is set by the size of the female as embryos develop to crawl-away juvenile snails inside an embryo-pouch under the shell of the female. To increase egg-numbers would require smaller egg-sizes that may trade-off against egg survival. Probably restricted by the size of the embryo-pouch, new-born snails are delivered at a more or less constant rate over the season. Consequently, juvenile females will become sexually mature at any time of the year, probably setting the stage for a year-round mating season.

In summary, we have shown here that in three species of littorinids, males actively track females by using a cue added to their mucous trail. We also showed that male *L. saxatilis* retain the same capacity, but fail to discriminate among trails of conspecific males and females. From this we conclude that female *L. saxatilis* have either permanently lost this cue or have the ability to optionally remove the cue under snail densities that are the norm for this species, resulting in observed frequencies of male-male pairings in the wild being exceptionally high in this species. We have also presented direct and indirect support for costs associated with mating and we argue that female *L. saxatilis* are less likely to be sperm-limited than females of low-density species of littorinids and therefore are selected for trying to decrease the number of costly matings. Hence an obvious reason why females no longer have a gender cue in the mucous is that removing this cue effectively reduce number of costly matings, reflecting a sexual conflict over mating frequency. Masking gender identity to obtain copulations is common in animals [32], [33], however evidence of females avoiding males by mimicking the male phenotype is scant. A few examples are found among species of damselflies, in which a proportion of females develop into andromorph females mimicking males in colour and behaviour thereby becoming less attractive to males and effectively reducing costs of male harassment and excessive matings [34], [35] (but see [36] for a slightly different interpretation). In damselflies, however, this polymorphism is frequency-dependent with a stable proportion of the females being andromorph. This is because males learn to recognize andromorph females if these are too common. In addition there is a cost of increased predation rate and risk of sperm limitation for andromorph females that must be balanced by the potential rewards of reduced matings (reviewed in 2). In *L. saxatilis*, however, to remove the gender-specific cue and produce neutral trails will most likely evolve with no additional cost for the females, hence there is no trade-off for the female snails and we predict that all of them have adopted the “gender neutral” strategy.
